# Use of alcohol and drugs by Norwegian employees: a pilot study using questionnaires and analysis of oral fluid

**DOI:** 10.1186/1745-6673-5-13

**Published:** 2010-06-15

**Authors:** Hallvard Gjerde, Asbjørg S Christophersen, Inger S Moan, Borghild Yttredal, J Michael Walsh, Per T Normann, Jørg Mørland

**Affiliations:** 1 Norwegian Institute of Public Health, PB 4404 Nydalen, NO-0403 Oslo, Norway; 2 Norwegian Institute for Alcohol and Drug Research - SIRUS, PB 565 Sentrum, NO-0105 Oslo, Norway; 3 The Walsh Group, 6701 Democracy Blvd, Suite 300, Bethesda, MD 20817, USA

## Abstract

**Background:**

The use of alcohol and drugs may affect workplace safety and productivity. Little is known about the magnitude of this problem in Norway.

**Methods:**

Employee recruitment methods with or without individual follow-up were compared. The employees filled in a questionnaire and provided a sample of oral fluid. Samples were analysed for alcohol, ethyl glucuronide (EtG; a biological marker of recent large alcohol intake), psychoactive medicinal drugs and illegal drugs.

**Results:**

Participation rates with and without individual follow-up were 96% and 68%, respectively. Alcohol was negative (≤0.1 mg/ml) in all samples, but 21.0% reported the intake of alcohol during the last 24 h. EtG was positive (>2.2 ng/ml) in 2.1% of the samples. In-efficiency or hangover at work during the past year was reported by 24.3%, while 6.2% had been absent from work due to the use of alcohol. The combination of self-report and analytical testing indicated that medicinal or illegal drugs had been used during the last 48 h by 5.1% and 1.7% of the participants, respectively; while only 4.2% and 0.4% admitted the use in the questionnaire.

**Conclusions:**

Self-reported data suggest that hangover after drinking alcohol appears to be the largest substance abuse problem at Norwegian workplaces, resulting in absence and inefficiency at work. Analysis of oral fluid revealed that the use of illegal drugs was more common than drinking alcohol before working or at the workplace. The analysis of oral fluid may be a valuable tool in obtaining additional information on alcohol and drug use compared to using questionnaires alone.

## Background

Alcohol use may affect workplace safety and productivity primarily in three ways: impairment due to the presence of alcohol in the blood, residual after-effects after large alcohol intake (in this report called "hang-over"), which also may result in short-term sick leave, and long-term sick leave due to chronic illnesses caused by alcohol abuse [1].

The use of psychoactive drugs may also affect safety and productivity. Approximately 20% of workers in Norway are using prescribed medicinal drugs with abuse potential, primarily sleeping agents, tranquillizers, and opioid analgesics [2]. The use of illegal drugs is less common in Norway than in the USA and some countries in southern Europe. In a Norwegian study published in 2001, only 2.7% of employees reported having used illegal drugs during the previous 12 months [3], while in a study performed in 2004, 2.2% of the general population reported the use of cannabis during last month according to the EMCDDA report for 2009 [4]. In comparison, about 7% of the general population in Spain and Italy reported the use of cannabis during last month [4], and 8.0% of American full-time workers and 10.2% of part-time workers reported the use of illegal drugs during last month according to the US national survey for 2008 [5].

Workplace drug testing (WDT) programmes were initiated in the USA in the 1980s to reduce alcohol and drug related accidents on the workplace [6]. Some companies experienced marked reduction in accidents after implementing WDT programmes [7]. Studies on productivity found that subjects testing positive for drugs used more sick leave, and had higher risks for being fired, and had higher turnover [8,9]. It was also claimed that WDT programmes would create drug-free workplaces which would improve job satisfaction [10]. However, other studies were not able to confirm the findings mentioned above. Today an important purpose of WDT programmes is to get substance abusing employees into treatment, provide the opportunity to get help, and to get the individuals back on the job [11].

WDT programmes are used for pre-employment testing (e.g., requiring urine samples from job applicants) and thus rejecting applicants being positive for illegal drugs, but also for post-employment surveillance (e.g., requiring urine samples from existing employees on a random, comprehensive, or suspicion basis) and for follow-up testing. The use of WDT is increasing in Europe, but is not as widespread as in the USA. In Europe WDT is mainly done in the transport, petrochemical, shipping, automobile, pharmaceutical and computer industry, and for call centres [12]. In Norway few companies have implemented this type of programme.

Normally, urine samples are used for WDT. The samples are most commonly tested for amphetamines, cannabinoids, cocaine, opiates and sometimes benzodiazepines and alcohol [12-14]. The finding of a drug or drug metabolite in urine will not indicate drug impairment at the time of sampling; it merely indicates that the drug has been used within a time period of several days or more prior to sampling, including use that does not directly affect working safety or productivity. Blood samples could be used to reveal possible alcohol and drug influence at the time of sampling. Furthermore, blood samples could also be used to look for biological markers of high alcohol consumption and might therefore give more information about the alcohol consumption during the last months [15,16]. Blood samples are rarely used in WDT programmes because taking a blood sample may be considered as a stronger invasion of privacy than taking a urine sample. Oral fluid may be used to detect and monitor recent use of alcohol (i.e. during the last 12 hours) and drugs (during the last 1-2 days), and the use of oral fluid testing in WDT has been increasing during the last years [17-20]. Oral fluid is an easily available medium that can be collected with non-invasive methods without the intrusion of privacy and with little chance of adulteration (unlike urine). Oral fluid has about the same detection time window as blood regarding alcohol and drugs, and can to some extent be a substitute for blood samples; oral fluid is probably the only other easily available body fluid that might parallel blood in some regards and may be related to behavioural performance [21]. The presence of drugs or drug metabolites in oral fluid indicate very recent drug intake [22], and reflects better than urine whether the subject may be impaired by drugs or alcohol at time of sampling [21].

Ethyl glucuronide (EtG) is a metabolite of alcohol that can be used as biological markers of recent alcohol intake [23-27]. EtG is usually measured in samples of urine or blood, but can also be detected in oral fluid [28]. High concentrations of EtG in oral fluid might reflect very recent intake of large amounts of alcohol even when no alcohol can be detected in the blood. However, the concentration of EtG in oral fluid, blood or urine does not reflect the alcohol consumption over a longer period of time and can therefore not be used to uncover excessive alcohol consumption or alcoholism. The kinetics of EtG in oral fluid compared to blood and urine has recently been published [29].

The aims of this pilot project were: to compare self-reported alcohol and drug use with results from analysis of oral fluid, to obtain information about the use of alcohol and drugs in the Norwegian workplace, and to examine some of the consequences that alcohol and drug use may have for sick leave, in-efficiency and hang-over at work. An additional aim was to compare two procedures for recruiting participants: one procedure with close contact between study personnel and employees and another procedure without.

## Methods

The procedures for recruitment of companies and employees was agreed upon in a meeting with representatives from The Norwegian Business and Industry Security Council, The Workplace Advisory Centre for Issues Related to Alcohol, Drugs and Addictive Gambling in the Workplace, and the Confederation of Norwegian Enterprises. The study was approved by the Regional Committee for Medical and Health Research Ethics.

### Subjects

Ten companies, research institutes and public administration bodies (hereafter called the "companies") were invited to participate in the project. In addition, advertisements were published in newsletters, newspapers and on websites, resulting in responses from two companies. In total, only four companies decided to participate. In addition, we collaborated with the Norwegian Transport Directorate at one checking station for heavy vehicles to include truck drivers.

The employees were recruited at the participating companies by first selecting one day for each company, then contacting all employees who either were present in the building or who passed the main entrance door during defined time periods. Employees were recruited within the first three hours after starting their work in the morning.

Occupational drivers were recruited at a checking station by first selecting two days, then contacting as many drivers as possible during two 2-hour periods within the timeframes of the scheduled heavy vehicle checks determined by the Norwegian Transport Directorate, covering both day and night.

Written and verbal information about the project was given, and participation was voluntary and anonymous.

Two approaches for recruiting participants were used, but only one method within each company.

### Recruitment procedure "A"

In some companies and for all occupational drivers, each employee was approached individually by one project assistant and asked to participate. An explanation about the project was given, and each person had the opportunity to ask questions. Those who agreed to participate completed a written questionnaire and provided an oral fluid specimen. The filled-in questionnaire and the sample of oral fluid were placed in a closed envelope and were collected by the project assistant either immediately of within short time of up to one hour.

### Recruitment procedure "B"

For other companies, an envelope containing the questionnaire and sampling kit for oral fluid, including instructions for use, were given to random employees when entering the company facilities in the morning. The employees were asked to deliver the questionnaire and the oral fluid sample in closed envelopes at specified sites before noon. They were given the phone number of a project assistant so that they could ask questions, if necessary.

### Sampling of oral fluid

Oral fluid was sampled using the Statsure Saliva Sampler™ (Statsure Diagnostic Systems, Framingham MA, USA), which had an indicator that turned blue when about 1.0 ml oral fluid had been collected. This device did not contain any saliva-stimulating agents. It was chosen because initial testing showed that samples collecting with this device were better suitable for the analysis of EtG than some other devices. For recruitment procedure "A", samples were chilled (about 2-8°C) for up to 10 hours, and thereafter kept frozen until the analyses were performed. For collection procedure "B", samples were kept at room temperature (about 22-25°C) for up to 5 hours before frozen.

### Analytical methods

Alcohol was determined by an enzymatic method [30]. The method was initially developed for the analysis of alcohol in blood and urine. Before we used it in this study, the method was validated for the analysis of alcohol in oral fluid. EtG and drugs were analysed by liquid chromatography - tandem mass spectroscopy [28,31]. The compounds analysed and the cut-off concentrations (in the mixture of oral fluid and buffer) are presented in Table 1. The cut-off concentrations were lower than those proposed by SAMHSA [32] in the USA because we wanted to detect all recent use of medicinal and illegal drugs to compare with self-reported drug use. The following drugs were defined as illegal: amphetamine, methamphetamine, MDA, MDEA, MDMA (ecstacy), cocaine, THC, and 6-monoacetylmorphine.

**Table 1:** Substances analysed and cut-off limits

Substance	Description	Cut-off
		ng/ml
Alcohol (ethanol)	Alcoholic beverages	0.1 mg/ml
Ethyl glucuronide	Metabolite of ethanol	2.2
Alprazolam	Benzodiazepine; anxiolytic	0.23
Amphetamine	Stimulant. Mostly used illegally in Norway	12.2
Benzoylecgonine	Degradation product and metabolite of cocaine	3.6
Carisoprodol	Muscle relaxant	13.0
Clonazepam	Benzodiazepine; anticonvulsant, anxiolytic	0.24
Cocaine	Stimulant; illegal in Norway	0.91
Codeine	Opiate analgesic, antitussive, antidiarrheal properties	3.7
Diazepam	Benzodiazepine; anxiolytic, anticonvulsant, sedative, skeletal muscle relaxant	0.18
Flunitrazepam	Benzodiazepine; hypnotic	0.16
Lorazepam	Benzodiazepine; sedative, hypnotic, muscle relaxant, anxiolytic, anticonvulsant	0.48
Methadone	Opiate analgesic. Treatment of heroin addiction	3.9
Methamphetamine	Stimulant. Illegal in Norway	7.5
Meprobamate	Metabolite of carisoprodol	10.9
Morphine	Opiate analgesic. Also metabolite of codeine and heroin	3.6
Nitrazepam	Benzodiazepine; hypnotic	0.21
Nordiazepam	Psychoactive metabolite of diazepam	0.34
Oxazepam	Benzodiazepine; anxiolytic, anticonvulsant, sedative skeletal muscle relaxant	2.4
Tetrahydrocannabinol	Cannabis, hashish, marijuana (THC)	0.31
Zolpidem	Short-acting non-benzodiazepine hypnotic	0.08
Zopiclone	Short-acting non-benzodiazepine hypnotic	0.49
3,4-methylenedioxy amphetamine	Illegal psychedelic hallucinogenic drug (MDA)	9.0
3,4-methylenedioxy-N-ethylamphetamine	Illegal psychedelic hallucinogenic drug (MDEA)	10.3
3,4-methylenedioxy-N-methylamphetamine	Illegal psychedelic hallucinogenic drug (MDMA)	1.9
6-monoacetylmorphine	Degradation product and metabolite of heroin, only illegal use in Norway	0.41
7-aminoclonazepam	Degradation product and metabolite of clonazepam	0.36
7-aminoflunitrazepam	Degradation product and metabolite of flunitrazepam	0.07
7-aminonitrazepam	Degradation product and metabolite of nitrazepam	0.31

### Statistical methods

Pearson's Chi-Square two-sided test for categorical variables was used for statistical evaluation of results in relation to age group by using SPSS 14.0 statistical software (SPSS Inc., Chicago, IL, USA).

## Results

A total of 526 employees participated in the study, representing research and development (n = 82), manufacturing and warehouse (n = 107), transportation (n = 126) and public administration (n = 211). Of these, 181 were women and 281 were men, while 64 subjects did not report the gender in the questionnaire. All age groups were represented. Two individuals did not provide samples of oral fluid, while seven did not fill in the questionnaire.

### Participation rates

The total participation rate was 82%. For the employees who were followed-up individually by a project assistant (recruitment procedure "A"), the participation rate was 96%, while when the employee was asked to deliver the questionnaire and sample in boxes at specified sites (recruitment procedure "B"), the participation rate was 68%.

### Age and gender

The distributions of age and gender were different for the companies studied. As a result of the fact that only one recruitment procedure was used in each company, and that few companies participated, the distribution of age and gender were different for the participants recruited with the two procedures. For recruitment procedure "A", 16.5% were females and 20.1% were below 30 years of age; while for recruitment procedure "B", 61.1% were females and 8.5% were below 30 years of age. Therefore, we have not sufficient data to perform a good comparison of men and women, different age groups, or different recruitment procedures.

### Results of questionnaire and analysis of oral fluid

The principal results of the questionnaire and analysis of oral fluid are presented in Table 2. Drug concentrations in oral fluid and possible explanations are presented in Table 3 for all cases where drugs were found.

**Table 2:** Positive responses from questionnaire and analysis of oral fluid

Questionnaire (n = 519)	n	%
Consumed alcohol within the last 24 h	109	21.0
Consumed 4 or more drinks within 24 h	10	1.9
Absent from work due to drinking alcohol during last 12 months	32	6.2
In-efficiency or hangover at work due to alcohol during last 12 months	126	24.3
Consumed 6 drinks or more in one session at least once a month	105	20.2
Deficient memory after drinking session at least once last 12 months	101	19.5
Deficient memory after drinking session at least once a month	6	1.2
Used medicinal drugs within 48 h	22	4.2
Absent from work because of using medicinal drugs during last 12 months	10	1.9
In-efficiency at work because of using medicinal drugs during last 12 months	17	3.3
Used illegal drugs within 48 h	2	0.4
Absent from work because of using illegal drugs during last 12 months	2	0.4
In-efficiency at work because of using illegal drugs during last 12 months	3	0.6

Analysis of oral fluid (*n *= 524)	*n*	%

Alcohol	0	0.0
Ethyl glucuronide	11	2.1
Medicinal drugs	14	2.7
Zopiclone	7	1.3
Zolpidem	2	0.4
Diazepam	4	0.8
Codeine	1	0.2
Illegal drugs	7	1.3
THC	5	1.0
Methamphetamine	2	0.4

Use of alcohol or drugs as revealed by oral fluid analysis and/or self-reporting (*n *= 526)	*n*	%

Alcohol within last 24 h^1^	114	21.7
Consumed 4 or more drinks during last 24 h^1^	18	3.4
Used medicinal drugs within last 48 h^2^	27	5.1
Used illegal drugs within last 48 h^2^	9	1.7

^1^Self-reported alcohol intake or EtG detected in oral fluid.

^2^Self-reported use or drug detected in oral fluid.

**Table 3:** Cases where drug was detected in oral fluid

Subject	Drug findings in oral fluid	Possible explanation
Female, <25 y	Zopiclone 3 ng/ml	Sleeping tablet the night before
Female, 35-39 y	Zopiclone 8 ng/ml	Sleeping tablet the night before
Female, 45-49 y	Zopiclone 23 ng/ml	Sleeping tablet the night before
Female, 50-54 y	Zopiclone 13 ng/ml	Sleeping tablet the night before
Female, 55-59 y	Zopiclone 13 ng/ml	Sleeping tablet the night before
Male, 60-64 y	Zopiclone 3 ng/ml	Sleeping tablet the night before
Male, 35-39 y	Zopiclone 47 ng/ml	Sleeping tablet the night before
Male, <25 y	Zolpidem 0.4 ng/ml	Sleeping tablet the night before
Male, 55-59 y	Zolpidem 1.2 ng/ml	Sleeping tablet the night before
Male, 50-54 y	Diazepam 1.0 ng/ml, nordiazepam 2.4 ng/ml	Normal therapeutic use
Male, 55-59 y	Diazepam 0.4 ng/ml	Normal therapeutic use
Male, 55-59 y	Codeine 137 ng/ml	Normal therapeutic use
Male, <25 y	THC 6 ng/ml	Cannabis use >6 h earlier
Male, 25-29 y	THC 1102 ng/ml	Recent cannabis use
Male, 30-34 y	THC 6 ng/ml	Cannabis use >6 h earlier
Male, 30-34 y	THC 17 ng/ml	Recent cannabis use
Male, 35-39 y	THC 294 ng/ml	Recent cannabis use
Male, 35-39 y	Methamphetamine 1067 ng/ml, amphetamine 188 ng/ml, diazepam 1.8 ng/ml	Recent intake of moderate doses, or large doses 1-2 days ago
Male, 40-44 y	Methamphetamine 14700 ng/ml, amphetamine 1052 ng/ml, diazepam 275 ng/ml	Recent intake of large doses

When using recruitment procedure "A", which had a participation rate of 96%, 0.7% reported the use of illegal drugs in the questionnaire, whereas such drugs were found in 2.2% of the oral fluid samples (results not shown). In total, 3.0% either reported use of illegal drugs or were found to be positive for illegal drugs when analysing oral fluid. Of those with procedure "B", none reported illegal drug use, and no oral fluid sample was positive for illegal drugs.

As the number of men recruited with procedure "A" was fairly high, a comparison of males below and above 35 years of age was performed, results are presented in Table 4.

**Table 4:** Drug and alcohol use and consequences of alcohol use among male employees followed up individually.

	Age <35 years n = 66 %	Age ≥35 years n = 151 %	p-value^1^
Consumed 6 drinks or more in one occasion at least once a month	36.9	18.5	0.005
Absent from work due to consumption of alcohol during last 12 months	13.4	3.3	0.005
In-efficiency or hangover at work due to alcohol during last 12 months	29.9	10.7	0.001
Deficient memory after drinking session at least once last year	29.2	16.0	0.027
Use of psychoactive medicinal drugs during the last 48 hours^2^	1.5	5.3	0.199
Use of illegal drugs during the last 48 hours^2^	6.1	2.0	0.118

^1^Chi square test for differences between age groups. ^2^Self-reported use or drug detected in oral fluid.

## Discussion

The primary aims of this pilot project were to compare self-reported use of alcohol and drugs with analytical results for oral fluid, to demonstrate the usefulness of EtG as a marker of large alcohol intake before a working session, to study the participation rate when using two procedures for the collection of questionnaires and samples of oral fluid, and to collect some preliminary data on alcohol and drug use in the workplace in Norway.

Only a few additional cases of alcohol use, as indicated by the presence of EtG, were detected when analysing oral fluid in addition to using a questionnaire. However, the detection of EtG indicates an alcohol consumption of more than six drinks the night before [29], and a large proportion of the EtG-positive subjects did not report an alcohol consumption of that magnitude. Thus, the incidence of large alcohol intake was under-reported in the questionnaires. This agrees with the finding in a previous study, where the self-reported alcohol consumption was found to be significantly lower than actual alcohol consumption [33].

The analysis of oral fluid revealed some additional drug users compared to self-reported use. The use of psychoactive medicinal drugs was reported by 4.2%, but additional 0.9% was found by analysing oral fluid. The use of illegal drugs was reported by only 0.4%, and additional 1.3% was found by analysing oral fluid. Thus, users of illegal drugs were much more reluctant to admitting drug use than users of alcohol and medicinal drugs. Similar results have been found in other studies [34,35]. Since about 20% of the invited employees refused to participate, the real fraction of employees that had used illegal drugs during the last 48 hours may probably be higher.

The participation rate was very high if each employee was met individually before and after the questionnaire was filled in and the sample of oral fluid was collected. If the employees were asked to return the questionnaire and sample in a container within a couple of hours for later pick-up by the project team, the participation rate was significantly lower.

The use of illegal drugs was most commonly detected when the participation rate was high. This may suggest that a larger number of employees who suspected that a sample of oral fluid would reveal the use of illegal drugs refused to participate when not being followed up individually by a project assistant. However, differences in gender and age distributions between companies using the two recruitment procedures may be contributing to the observed difference.

In our study of male workers using procedure "A", both the incidences of binge drinking (more than 6 drinks in one occasion), absence from work, hangover and deficient memory were significantly higher among men below 35 years of age compared to older men. Similar result were found in a previous study [2]. The use of illegal drugs seemed also to be more prevalent among younger men, although not statistically significant; however, in a previous survey among random drivers, the highest prevalence of illegal drugs was also seen among young male drivers [36]. The use of psychoactive medicinal drugs seemed to be more prevalent among men at an age of 35 or higher. Similar results were found among drivers [36]. Data from the Norwegian Prescription Database [37] confirms that the use of psychoactive medicinal drugs is more common among older men.

There is a large variation in drug concentrations ratios between oral fluid and blood, so blood drug concentrations cannot be accurately estimated by using concentrations in oral fluid [38]. Drug concentrations in oral fluid can neither be used to assess psychomotor impairment nor assess whether decision-making is affected. However, the drug findings may be used to suggest whether or not a person has used a normal therapeutic dose of a medicinal drug, or whether or not the use of an illegal drug happened shortly before sampling. The concentrations of medicinal drugs found in our study suggested that normal therapeutic doses of medicinal drugs had been used, except in one case where also methamphetamine had been taken.

A combination of methamphetamine and diazepam was found in two samples. In those cases, amphetamine was also detected as a metabolite of methamphetamine. The combination of methamphetamine and sedatives may indicate chronic addictive methamphetamine use. The detection time frame for methamphetamine in oral fluid has been reported to be about 24 hours [39], but might be longer after intake of large doses. In one case the concentrations of both methamphetamine and diazepam were very high, indicating very recent intake of large doses. In the second case, the intake may have happened 1-2 days ago, or a smaller dose might have been taken recently. The abuse of amphetamines should be a serious concern for their employers.

The detection time frame for THC in oral fluid is about 34 hours [40]. We found both very high and fairly low concentrations of THC in our study. In a study performed by Kauert and co-workers, THC concentrations of 18 ± 12 ng/ml were observed 6 hours after smoking cannabis [41]. Similar results were observed in another study [42]. Based on these results, it is likely that three of the five subjects with positive THC findings in our study had smoked cannabis within 6 hours before sampling of oral fluid.

It seems likely that three to five of the seven workers who had used illegal drugs had probably reduced working performance at the time of sampling of oral fluid.

EtG was found in samples from 11 subjects. Knowing that an alcohol intake corresponding to six drinks or more is needed in order to give detectable concentration of EtG in oral fluid about 12 hours after start of drinking [29], we may assume that a proportion of those subjects experienced hangover at work at the time of oral fluid sampling. Hangover after alcohol use may be a more significant reason for reduced performance among Norwegian employees than alcohol impairment at work. This is also in compliance with the self-reported inefficiency or hangover at work using the questionnaire.

## Conclusions

Our findings suggest that analysis of oral fluid may be a valuable tool in obtaining supplementary information when performing studies on the use of alcohol and drugs with questionnaires, particularly regarding the use of illegal drugs, which was very much under-reported. Our findings also suggest that the use of drugs may be more common than drinking alcohol prior to working or at the workplace. Self-reported data suggest that hangover after alcohol use may be a more significant problem at the workplace than impairment by alcohol or drugs at work.

## Competing interests

The authors declare that they have no competing interests.

## Authors' contributions

All authors participated in designing the study and interpreting the data. BY had the main responsibility for planning and coordinating the acquisition of data. HG had the main responsibility for drafting the manuscript, and all coauthors contributed in revising it critically for intellectual content. All authors read and approved the manuscript.
